# Prioritizing a Research Agenda of Transitional Care Interventions for Childhood-Onset Disabilities

**DOI:** 10.3389/fped.2021.682078

**Published:** 2021-09-13

**Authors:** Andrea Duncan, Dorothy Luong, Laure Perrier, Mark T. Bayley, Gail Andrew, Kelly Arbour-Nicitopoulos, Brian Chan, C. J. Curran, Gina Dimitropoulos, Laura Hartman, Lennox Huang, Monika Kastner, Shauna Kingsnorth, Anna McCormick, Michelle Nelson, David Nicholas, Melanie Penner, Laura Thompson, Alene Toulany, Amanda Woo, Joanne Zee, Sarah E. P. Munce

**Affiliations:** ^1^Institute of Health Policy, Management and Evaluation, University of Toronto, Toronto, ON, Canada; ^2^Occupational Science and Occupational Therapy, University of Toronto, Toronto, ON, Canada; ^3^Toronto Rehabilitation Institute, Toronto, ON, Canada; ^4^University of Toronto Libraries, University of Toronto, Toronto, ON, Canada; ^5^Division of Physical Medicine, University of Toronto, Toronto, ON, Canada; ^6^Department of Pediatrics, University of Alberta, Edmonton, AB, Canada; ^7^Faculty of Kinesiology and Physical Education, University of Toronto, Toronto, ON, Canada; ^8^Holland Bloorview Kids Rehabilitation Hospital, Toronto, ON, Canada; ^9^Faculty of Social Work, University of Calgary, Calgary, AB, Canada; ^10^Department of Paediatrics, Faculty of Medicine, University of Toronto, Toronto, ON, Canada; ^11^The Hospital for Sick Children, Toronto, ON, Canada; ^12^Department of Family and Community Medicine, Faculty of Medicine, University of Toronto, Toronto, ON, Canada; ^13^North York General Hospital, Toronto, ON, Canada; ^14^Rehabilitation Sciences Institute, University of Toronto, Toronto, ON, Canada; ^15^Department of Pediatrics, University of Ottawa, Ottawa, ON, Canada; ^16^Children's Hospital of Eastern Ontario, Ottawa, ON, Canada; ^17^Sinai Health System, Toronto, ON, Canada

**Keywords:** transitional care, intervention prioritization, childhood, disability, modified Delphi

## Abstract

Transitional care interventions have the potential to optimize continuity of care, improve health outcomes and enhance quality of life for adolescents and young adults living with chronic childhood-onset disabilities, including neurodevelopmental disorders, as they transition to adult health and social care services. The paucity of research in this area poses challenges in identifying and implementing interventions for research, evaluation and implementation. The purpose of this project was to advance this research agenda by identifying the transitional care interventions from the scientific literature and prioritize interventions for study. A modified-Delphi approach involving two rounds of online surveys followed by a face-to-face consensus meeting with knowledge users, researchers and clinician experts in transitional care (n = 19) was used. A subsequent virtual meeting concluded the formulation of next steps. Experts rated 16 categories of interventions, derived from a systematic review, on importance, impact, and feasibility. Seven of the 16 interventions categories received a mean score rating of ≥7 (out of 10) on all three rating categories. Participants then rank ordered the reduced list of seven interventions in order of priority and the top four ranked interventions advanced for further discussion at a consensus meeting. Using the Template for Intervention Description and Replication (TIDieR) checklist as a guide, the participants identified that a study of a *peer system navigator* was worthy of future evaluation. This study highlighted that transitional care interventions are complex and multifaceted. However, the presence of a peer to support system navigation, advocacy and individual and family education was considered the most ideal intervention addressing the current gap in care. Future research, which aims to engage patients and families in a co-design approach, is recommended to further develop this intervention.

## Introduction

Youth living with childhood-onset disabilities may experience challenges during the transition process from pediatric to adult health and social services. Adolescence in itself is an important period of transition involving multiple changes and choices related to identity, sexuality, education and work (1). Living with chronic disabilities can further complicate this developmental period. The process of transition to adult care is often complex, multifaceted and maintaining continuity of care requires considerations beyond the medical needs and preparation for new healthcare environments (2, 3). Adolescents and young adults with disabilities are at higher risk of poor mental health outcomes such as anxiety disorders, depression, suicidal ideation, and suicide attempts (4–9). As adults, they face the risk of restricted participation in many aspects of community integration such as housing, intimate relationships and employment (10). Psychosocial, educational, residential, vocational and recreational needs are therefore important factors to consider during transition, and have more recently been recognized as important determinants of health and critical components of a holistic strategy for healthcare transition (2, 11). Without multi-faceted and multi-disciplinary approaches, negative outcomes can include diminished quality of life and unnecessary stress on healthcare systems (12–14).

Transitional care interventions (TCIs) are a wide range of services and interventions known to promote continuity of care, reduce significant detrimental physical and mental health outcomes, and improve the quality of life of adolescents and young adults living with childhood-onset disabilities as they age. Examples of TCIs include preparing patients and families for transition readiness, promoting information continuity amongst health professionals or case management services through the transition process (15). The paucity of research in this area poses challenges in terms of identifying and prioritizing interventions to implement and evaluate. Where evidence does exist on TCIs, most evaluation studies are descriptive in nature (15), and are lacking in rigorous design (16), valid and reliable instruments for evaluation (17) and external validity (18). Additionally, the high variability across practice settings and the siloed nature of health and social services have led to issues with reliability and transferability across settings and contexts (15). These considerations leave researchers and clinicians struggling to identify the best next steps in the selection, implementation and evaluation of TCIs for adolescents and young adults with childhood-onset disabilities. To address this gap in knowledge, our objective was to prioritize the TCIs found in the literature and then identify one intervention best suited for a future relevant, high-quality, and holistic research agenda.

## Methods

We conducted a modified Delphi (19) with knowledge users, researchers, and clinician experts in transitional care to narrow a set of previously identified TCIs (15). Specifically, our process included two rounds of online surveys, one large group face to face consensus meeting and a virtual planning meeting to prioritize TCIs for future evaluation and create a plan for execution. This study was approved by the University Health Network Research Ethics Board (REB 19-5746) and all participants provided informed consent prior to taking part in the study.

### Participant Recruitment

A snowball sampling approach (20) was used to recruit local, national and international experts in transitional care for youth with childhood onset disabilities. Following recommended practices in Delphi methodology for size of expert groups (21), and to account for potential attrition, we recruited participants on a rolling basis with an aim of approximately 20 and achieved participant diversity in discipline, role and region.

### Modified Delphi Approach

The Delphi method is a process whereby multiple rounds of feedback from a group of experts are solicited (22). Repeated surveys are conducted and after each round, responses are aggregated and shared with the group before the next round. This allows experts to adjust their answer based on how they interpret the group response, and the final outcome is meant to reflect a true group consensus (22). The *modified* Delphi mirrors the regular Delphi in using repeated surveys to arrive at consensus. Where it differs from convention is that it begins the process with pre-selected items drawn from earlier work, rather than using the experts to brainstorm on a particular subject (23). In our modified Delphi approach, two rounds of feedback were conducted. The surveys were informed by a systematic review conducted by the research team, predominantly focused on neurodevelopmental disorders and their associated complexities (15). The face-to-face consensus meeting was convened to prioritize one intervention for future evaluation. A core research group met virtually to further plan the implementation of a future evaluation.

### Modified Delphi Surveys

Both survey rounds were conducted using *Hosted in Canada Surveys* (https://www.hostedincanadasurveys.ca). Consistent with healthcare-oriented modified-Delphi processes, the multi-round surveys employed rating and ranking exercises (24).

In Round 1, experts were presented with a list of 16 categories of TCIs, based on a systematic review conducted by the research team (15). Experts were asked to rate their level of agreement on a Likert-scale from 0 = strongly disagree to 10 = strongly agree on statements related to importance, impact of the intervention, and feasibility of the intervention for a future evaluation. We deemed these criteria as central to developing an actionable research agenda. Experts were also given the option to comment on the particular interventions listed and add intervention categories not identified in the list. Mean scores out of 10 for each statement, under each of the interventions were calculated and open-ended answers were scanned to identify any new interventions. An a-priori cut-off mean score of >7 on each rating category was set to narrow the list and move a subset of interventions to Round 2.

In Round 2, experts were presented with the condensed list of interventions and asked to rank order from most to least important. As with Round 1, experts were asked to comment on the particular interventions listed and add any other intervention categories not identified. A total score for each intervention listed in the Round 2 survey was calculated by assigning a reverse weighting. Each experts' rank order was scored and all scores for a particular intervention were summed to create an overall score for that intervention (25). Open-ended answers were scanned to identify new intervention categories as well as identify issues to consider regarding the prioritization of particular interventions.

### Consensus Meeting

We then hosted a face-to-face, 1.5-day consensus meeting to serve as an opportunity to deliberate and finalize the top priorities for TCIs identified from the modified Delphi, as well as to support discussion in developing a research agenda. A facilitator with expertise in group facilitation and extensive knowledge of healthcare delivery systems supported the consensus meeting. A research assistant captured all key discussion points in meeting notes.

The meeting commenced with a presentation of the results of the systematic review that informed the modified Delphi process followed by a presentation of the results of the two rounds of surveys for the modified Delphi. A recorded presentation from a young adult with cerebral palsy and lived experience with transitional care challenges was shared to highlight the importance of first-person considerations.

Experts discussed and developed consensus for the most significant TCIs. Specifically, the meeting focused on defining the top four TCIs identified in the modified Delphi, discussing the implementation considerations of these interventions, and devising a priority research agenda and key steps for its advancement. Specifically, the Template for Intervention Description and Replication (TIDieR) checklist and guide (26) was used as a tool to structure discussions and ensure the components of the interventions were comprehensively documented. In small groups, meeting participants described items 1 to 7 on the TIDieR checklist of one intervention. These components were then presented back to the larger group for discussion. The small groups then switched interventions and further described items 8 to 12 on the TIDieR checklist of the intervention. Large group discussions were repeated followed by reflecting on optimal research questions for their assigned intervention based on the four interventions. The consensus meeting concluded with discussing a focused research agenda based on one intervention.

### Virtual Research Planning Meeting

Lastly, with a priority TCI identified, a virtual meeting was held amongst experts who indicated an interest in further discussing and refining the proposed research including study focus and methods. The experts were provided with a summary of the consensus meeting and then participated in a consensus discussion around next steps for a study of the prioritized intervention.

## Results

In total, 19 out of 29 invited experts in TCIs consented to participate in the modified Delphi process. This included individuals working within pediatric and adult specialty care medicine, psychology, social work, occupational therapy, physical therapy, kinesiology, therapeutic recreation and nursing; individuals working in appropriate health, social, and non-governmental organizations; and, researchers with expertise in transitional care for adolescents and young adults with childhood-onset disabilities, health services research and knowledge translation. The majority of experts were female (84%) and included healthcare professionals, researchers, and those with a combined role of researcher and healthcare professional. More than half of the experts (63%) indicated 20 or more years of experience in their area of expertise, with over one third of respondents (42%) specifying expertise in pediatrics.

In the Round 1 rating survey, all interventions received a mean score rating of ≥7 on importance and impact; however, only seven of the interventions received a mean score rating of ≥7 on the third rating category related to feasibility. See Supplementary Table 1 for a list of the interventions, components and their associated scores. The experts did not add any new interventions to the list for consideration.

In the Round 2 ranking survey, participants were asked to rank and prioritize the 7 TCIs identified in Round 1. See Supplementary Table 2 for these results.

### Consensus Meeting

The experts indicated that it was important to focus on the top four TCIs identified in the surveys. See Supplementary Table 3 for the specific TCI descriptions developed by the smaller working groups through the TIDieR checklist. As evident in the descriptions of the four TCIs, there was a great deal of overlap in terms of content and approach between these interventions. Although the consensus meeting sought to address the ambiguity amongst the various TCIs, the large group discussions highlighted that it was difficult to consider each intervention in isolation. Participants emphasized various reasons for not focusing on just one of the interventions under consideration; such as, the siloed nature of health and social systems, the complexity of patient and family needs and issues with communication between child and adult systems. Additionally, participants demonstrated highly varied views on the key drivers of success in transitional care, such as supportive family, naturally integrated systems and health professionals' readiness to support the transitional process. Despite efforts to converge and focus the discussion, participants often had “just one more comment” or other important items for consideration. The large group consensus was that addressing the key/active components of TCIs is like asking “what's in the stew” and that high flexibility amongst approaches is required. Medical complexity and social determinants of health were most referenced as issues that complicate the transition process.

After in-depth consultation and facilitated discussion, it became apparent that “case management” continued to arise as the most significant construct, as it was represented in all of the top four TCIs. While this was an intervention that scored low on the feasibility criteria in Round 1, after discussion, the participants agreed that the presence of a “coach,” “peer” or “system navigator” should be the key intervention of a future research study. Specifically, participants agreed that evaluating the impact of “a person” who would act as an educator, advocate and system navigator to support patients and families through the transition into the adult system would best serve the population. Issues of concern remained about comparison groups, population, geography and identity of the case manager.

### Virtual Research Planning Meeting

Although not initially planned as part of the modified Delphi process, we added a final, brief virtual meeting with interested participants who held research roles and expertise to build a more focused evaluation plan. While the consensus meeting was successful at prioritizing one TCI for future research, a focused research discussion to plan an optimal future evaluation was warranted.

It was agreed upon by all the researchers that a targeted intervention using peers with lived experience of transitioning into the adult system as peer navigators offered a promising approach and a unique future evaluation opportunity. A proposed intervention described using the TIDieR checklist, was developed during this discussion (see Supplementary Table 4).

The researchers also determined that this research agenda would benefit from input and participation from patients and families. This notion initially arose during the consensus meeting; however, was reinforced in the final research discussion. It was determined that the next steps in the research development plan would require a co-design approach with these partners.

## Discussion

Using the modified Delphi approach, we sought the opinions of experts on TCIs for adolescents and young adults with childhood-onset disabilities, including neurodevelopmental disorders, with the intention of prioritizing key components for future implementation and evaluation. The results of our previous systemic review identified a list of 16 interventions (15) that were prioritized by transitional care experts to four interventions after two survey rounds and then one intervention of “peer system navigator” after two consensus meetings.

Collaborative discussions with experts highlighted that TCIs for adolescents and young adults with childhood-onset disabilities are complex and multifaceted. Using the TIDieR framework to focus the discussions, experts indicated that the presence of an individual playing the role of system navigator, educator, advocate, coach and case manager would be an ideal intervention for further evaluation. More specifically, a focused evaluation of trained peer transitional coaches is needed to advance the evidence in TCIs for youth and young adults with childhood-onset disabilities. The notion of a peer system navigator is still unique to this population.

Our findings are consistent with other researchers in the field of pediatric TCIs. Dimitropoulos et al. (27) previously highlighted that children with complex health needs benefit from a care coordinator to promote transition but indicated that the title and scope of this navigator is varied in the literature and practice. Through interviews with health professionals in the field, the researchers identified that the process of transitional navigation should embrace a four-stage process including: “(1) identification of young people with special healthcare needs and families requiring support, (2) preparation for transfer, (3) health system navigation and (4) post-transfer support.” In general, we recognize that a transitional care coordinator can have benefits for patients, their families and the entire healthcare system; however, we also recognize that healthcare providers have traditionally assumed these roles.

Our study had many strengths. First, to our knowledge, there are no studies assessing the impact of a peer transition coach to assist the population of youth with childhood-onset disabilities as they transition to the adult system. The role of a “peer” has gained increasing recognition in the literature and practice amongst other populations. The literature on peer support suggests that peers can help patients develop improved well-being while decreasing symptoms and hospitalizations (28). Furthermore, peers are known to provide valuable support across varying conditions such as diabetes (29), mental health (30), and cancer (31). Lastly, findings from more recent evidence have demonstrated that peers can also play an impactful role in supporting the system navigation process (32–34).

Based on these findings, we propose that peers could potentially fulfill an important role as transitional care coaches within the population of youth with childhood-onset disabilities transitioning to the adult care system and community. The collective results of the systematic review, modified Delphi process and consensus meetings have culminated in an emergent understanding that system navigation lead by peers is a potential impactful intervention worthy of further investigation for this population, and that there is a unique opportunity to train adults who have successfully transitioned to the adult system to support patients and their families with this same process. We anticipate that peers may be well-suited for the provision of “health system navigation” and “post-transfer support” (27), yet further systematic examination is needed.

Before proceeding to such an investigation, key considerations seem warranted. Firstly, prior to potentially considering specific activities, processes and competencies to be integrated in the role of peer navigator, the perspectives and preferences of patients and families need to be sought. As such, a co-design approach is needed to elicit the perspectives of patients' and families' and ensure that they inform the nuanced elements of the implementation and evaluation protocols. Additionally, the need to create core competencies of a peer system navigator has been emphasized in the literature (30, 32), and this will need to be addressed and outlined before an evaluation begins. Baumann and Christophilakis (unpublished data), conducted semi-structured interviews with young adults with childhood onset disabilities and determined that some of the components of this training should include advocacy, empathy, interpersonal communication, motivation and goal setting and self-management skills.

With a concrete construct identified, and clear vision on the next steps of building the research protocol with a co-designed approach, we are confident that a solid evaluation structure is being established. Both quantitative and qualitative approaches will be necessary to evaluate the efficacy/effectiveness of such a targeted intervention. We are optimistic that this essential evaluation will be attractive to future granting agencies.

## Study Limitations

We acknowledge study limitations. Although we followed modified Delphi practices based on the precedent of previous authors (25), real time adjustment of modified Delphi practices were required to reach our objective of developing a focused evaluation plan for one TCI. The complexity of TCIs and the variability in the population's health, social and geographic considerations are thought to be the reason for these necessary methodological adjustments. We are unaware of other researchers who have articulated struggles with group consensus at the final stage of the modified Delphi process; however, we speculate that we are not the first research group to experience this phenomenon. Acknowledging this challenge was therefore judged to be an important addition to the current paper. However, we also acknowledge that a different mix of stakeholders, a different set of activities for the consensus meeting or an alternate approach to facilitation may have led to a different result.

## Conclusions

This study commenced with 16 potential TCIs for adolescents and young adults who have a childhood-onset disability and are transitioning to the adult healthcare system. Using a modified Delphi process, we were able to prioritize one key intervention and research agenda which involves the development and evaluation of a peer transitional coach. The next steps of our research agenda will be to engage patients and families in a co-design approach to optimize its function in the lives of the individuals it will endeavor to enable.

## Data Availability Statement

The original contributions presented in the study are included in the article/Supplementary Material, further inquiries can be directed to the corresponding author/s.

## Ethics Statement

This study involved human participants and was reviewed and approved by University Health Network Research Ethics Board (REB 19-5746). The patients/participants provided their written informed consent to participate in this study.

## Author Contributions

AD and DL: led the creation of this manuscript. SM: research project and manuscript creation. All authors contributed to the creation of the grant, study methodology, data analysis, and review and feedback of the manuscript.

## Conflict of Interest

The authors declare that the research was conducted in the absence of any commercial or financial relationships that could be construed as a potential conflict of interest.

## Publisher's Note

All claims expressed in this article are solely those of the authors and do not necessarily represent those of their affiliated organizations, or those of the publisher, the editors and the reviewers. Any product that may be evaluated in this article, or claim that may be made by its manufacturer, is not guaranteed or endorsed by the publisher.
