# Supplementary material for: Prioritizing a Research Agenda of Transitional Care Interventions for Childhood-Onset Disabilities
Source: Front Pediatr. 2021 Sep 13;9:682078. doi: 10.3389/fped.2021.682078 (PMC8475648; doi:10.3389/fped.2021.682078)
Supplement: Supplementary file 1 [file Table_1.DOCX]

Supplementary Material

# Supplementary Tables

**Table 1. Delphi Round 1 Results**

| **Transitional Care Interventions** | **Mean Score Rating out of 10 (SD)**  **n=17** | | |
| --- | --- | --- | --- |
|  | **Overall Importance** | **Impact on Quality of Life** | **Feasibility of Implementation** |
| Interventions that focus on transition readiness, self-determination and self-advocacy for young people with disabilities * | 8.41 (2.18) | 8.00 (2.60) | 7.41 (2.12) |
| Promoting consistent management of the health condition with decision support tools: Embedded service delivery tools to help healthcare and other professionals with the transition process e.g., summary documents with pre-identified information, linking services, checklists and prompts to discuss transition as transition approaches * | 8.47 (2.53) | 7.94 (2.72) | 7.29 (2.91) |
| Promoting information continuity for health professionals with multi-source transfer summary of young person with disabilities’ transition needs: e.g., comprehensive amalgamation of young person’s health reports from various healthcare providers, providing an overall summary of young person’s transition needs * | 8.47 (2.62) | 7.88 (2.71) | 7.59 (2.60) |
| Transition care education for healthcare professionals: e.g., educational interventions related to transition for current healthcare professionals or healthcare professionals in training * | 8.71 (2.41) | 7.64 (3.37) | 8.35 (1.77) |
| Toolkits for healthcare professionals on the best practices of transition: e.g., tools and interventions that can be implemented by healthcare or other professionals working with young people with disabilities, such as individual self-management plans or transition protocols * | 8.64 (1.87) | 7.88 (1.93) | 7.76 (1.68) |
| General transition education for young people with disabilities and their families* | 7.76 (2.86) | 7.21 (2.81) | 7.94 (2.73) |
| Parent education on how to advocate for adult disability services* | 7.53 (2.96) | 7.24 (2.88) | 7.65 (2.71) |
| Dedicated transition clinics/transition teams: multidisciplinary clinics/teams working solely on transition for young people with disabilities and providing direct support to young people with disabilities and their families | 7.88 (2.89) | 7.94 (2.81) | 6.00 (2.95) |
| Case management or case coordinator model: dedicated transition care coordinator/case manager working with young people and their families to develop individualized transition plans | 8.58 (2.47) | 8.35 (2.37) | 6.64 (2.15) |
| Peer Mentoring programs aimed at young people with disabilities: expose/offer young people with disabilities with examples of/contact with successful adults who are persons with similar disabilities | 7.59 (2.53) | 7.41 (2.71) | 6.76 (2.80) |
| Independent living skills/self-management training programs for young people with disabilities | 8.06 (2.56) | 8.18 (2.56) | 6.88 (2.59) |
| Vocational skills training programs for young people with disabilities | 7.88 (2.12) | 7.76 (2.11) | 6.29 (1.99) |
| Extended transition services: interventions that extend beyond initial skills/self-management/vocational/self-determination and self-advocacy training aimed at young people with disabilities; focused on helping young people to continue building the skills that were taught in initial interventions | 8.47 (2.65) | 8.29 (2.54) | 6.29 (2.17) |
| Consultation interventions: e.g., Consultant working with young person with disabilities, caregiver, and teacher in school settings to provide coaching sessions related to identifying young person’s goals, strategies to meet goals, and problem-solving related to accomplishment of goals | 7.94 (2.46) | 7.88 (2.50) | 6.06 (2.61) |
| Teacher education about transitions for young people with disabilities | 7.76 (2.75) | 7.41 (3.20) | 6.59 (2.91) |
| Employer education about transitions for young people with disabilities | 7.00 (3.78) | 7.18 (3.23) | 5.41 (3.12) |

**Interventions with a mean score of at least 7/10 on all three rating categories*

**Table 2. Final Ranking of the Top Seven Transitional Care Interventions, as Determined by the Modified Delphi Expert Panel**

| Intervention | Frequency of Occurrence *  Rank (score) | Total Score | Rank Order |
| --- | --- | --- | --- |
| Interventions that focus on transition readiness, self-determination and self-advocacy for young people with disabilities | 1 (7) = 11  2 (6) = 1  3 (5) = 4  4 (3) = 1  3 (2) = 0  2 (1) = 1  No rank (1) = 1 | 110 | 1 |
| Promoting consistent management of the health condition with decision support tools: Embedded service delivery tools to help healthcare and other professionals with the transition process e.g., summary documents with pre-identified information, linking services, checklists and prompts to discuss transition as transition approaches | 1 (7) = 1  2 (6) = 7  3 (5) = 2  4 (3) = 4  3 (2) = 1  2 (1) = 2  No rank (1) = 2 | 84 | 2 |
| Promoting information continuity for health professionals with multi-source transfer summary of young person with disabilities’ transition needs: e.g., comprehensive amalgamation of young person’s health reports from various healthcare providers, providing an overall summary of young person’s transition needs | 1 (7) = 3  2 (6) = 3  3 (5) = 4  4 (3) = 2  3 (2) = 3  2 (1) = 1  No rank (1) = 3 | 81 | 3 |
| Transition care education for healthcare professionals: e.g., educational interventions related to transition for current healthcare professionals or healthcare professionals in training * | 1 (7) = 2  2 (6) = 2  3 (5) = 1  4 (3) = 3  3 (2) = 5  2 (1) = 3  No rank (1) = 3 | 67 | 4 |
| Toolkits for healthcare professionals on the best practices of transition: e.g., tools and interventions that can be implemented by healthcare or other professionals working with young people with disabilities, such as individual self-management plans or transition protocols | 1 (7) = 1  2 (6) = 1  3 (5) = 3  4 (3) = 4  3 (2) = 1  2 (1) = 5  No rank (1) = 4 | 61 | 5 |
| General transition education for young people with disabilities and their families | 1 (7) = 0  2 (6) = 3  3 (5) = 2  4 (3) = 2  3 (2) = 5  2 (1) = 3  No rank (1) = 4 | 61 | 6 |
| Parent education on how to advocate for adult disability services | 1 (7) = 1  2 (6) = 1  3 (5) = 3  4 (3) = 3  3 (2) = 2  2 (1) = 2  No rank (1) = 7 | 57 | 7 |

Scores were assigned on the basis of individual expert ranking and summated for a total score; rank order is based on total score.

*Numbers reported indicate the number of respondents selecting a particular ranking for an item.

**Table 3. – Results of TIDieR (Checklist Small Working Group)**

| Brief name | Interventions focused on transition readiness, self-determination and self-advocacy for young people with disabilities | Interventions focused on promoting consistent management of the health condition using embedded service delivery tools to help healthcare and other professionals with the transition process    Group renamed: Universal transition pathways to optimize decision making | Interventions focused on promoting information continuity for health professionals, particularly around sharing transitional needs between the transitioning person’s multiple healthcare  Group renamed: My Transition Summary | Interventions focused on transitional care education for healthcare professionals (TRACE)  Group renamed: TRACE program |
| --- | --- | --- | --- | --- |
| Why | Want to build capacity for individuals and families to develop skills to navigate the system. The system is less flexible to change | To establish a minimum standard of care; prevent “critical events” (e.g., keep people out of emergency department). Also to respond to knowledge and priority gaps | Person-centred care. Allows for reduction of repetition. Reduces anxiety amongst healthcare professionals | Not addressed |
| What | Assess readiness, skills and competency. Procedures focused on core skill development. System of customizable goals. | Lifespan care pathways with universal availability; technology can assist by identifying flags for risk, clear dates and milestones. Procedures focused on electronic medical records prompts and case management | Use of electronic, app that can be personalized. Issues of who owns it can be difficult to define. Time-stamped and alerts built in for pivotal times. Needs to be multilingual and culturally sensitive. | Transitional toolkit. Educating HCP and families and communities. Build into the EMR.  Bidirectional communication between paediatric and adult care. |
| Who provided | Health care providers. Also consider community service, mentors and peers | Not addressed by group | Systems level training; healthcare team and potentially others | Multidisciplinary |
| How | Training and simulation to develop skills | Invest more in adult care providers and improve primary care training. Building teams with tech experts, mental health support, care coordinators. System should be linked to paediatric and adult sides with remote and 24/7 access for crisis/questions. Technology “with heart” | 1 case conference mandated to make sure basic model is done by the time transition will happen | Use an EMR system with automated prompts. Questionnaires in patient portal system |
| Where | Not addressed by any group | | | |
| Large group discussion – identification of key important features of the intervention | Assessment, inclusion of families, term “coaching”, focus on practical deliverables, important to assess patient competency to manage vs self-report, developing skills for finding information | Mental health piece; technology (but balance); pathways/optimization; need to highlight the elements that are minimal vs optimal levels; tools and support to adult providers | Mandated real-time communication between providers; who owns document - sharing with young person and family; opportunities for personalization | Bi-directional communication; transition and nursing/HCP – it’s an ongoing process, not just an event; challenge we will always face is time; broader community -pathway to reflect that is important |
| When and how much | Start with transition assessment (physician, navigator, whoever – depending on where you live) | Tailored three to five-year intervention: Intervention 15-20; assigned a navigator, assess goal, work with community agencies | My transition summary is more about an “event” than just the process of transitioning | Start at age 12 |
| Tailoring | Set goals and a plan. Continually reiterate end point. Prioritize goals.  Consider age 14 to start. | Intensity of relationship/coordinator should be proportional to need | Start with EMR, connects speciality clinics and family doctors. =High variability of success across populations | Find balance between minimal to optimal |
| Modifications | Annual assessments until final assessment “safe” to transition.  Annual assessments are chance to adjust goals.  Layer in motivational interviewing? | High need = social determinants, not health condition | Want to be able to view info in EMR and add to it; patients have to be able to have access to it  Connect this to an app “My transition” app already exists – embed tools in it to link to own chart | High users vs low need patients |
| How well | Implement? A measure of transition of knowledge into practice. | Adherence to guideline – meet targets  Critical events  Patient reported quality of life  Health care utilization  Informal social support | So, what is that transition summary? What needs to be in the summary?  Compare success of arriving in the adult system | Self-management abilities. |

**Table 4 – TIDieR Checklist for an Evaluable Intervention**

| Brief name | Transitional Care Peer Navigation |
| --- | --- |
| Why | The system is siloed and fragmented. Families and patients need help with system navigation. Health care professionals are not addressing transitions and there are limited funds to add health care professionals as system navigators. Transitional care currently in place is focused on disease specific interventions and may not address quality of life, family support and transition to community. |
| What | Focused on goal setting around individual outcomes of interest (e.g., community integration) and assistance with compiling resources. One-on-one aspect would allow for tailoring of intervention. This approach would not be focused on one population, but would target a population (e.g., cerebral palsy) who has little support. |
| Who provided | Trained peers with lived experience transitioning to the adult system. |
| How | This pilot program would be focused on training peers on how to deliver this intervention. Trained peers would then meet with their peers to assess key, anticipated transition issues. A plan would be developed; plan and monitoring would take place until age 20. |
| Where | Face to face, telephone and virtual platforms are all deemed appropriate. |
| When and how much | Start at age 12 to 14. Minimum once a year up to four times a year. |
| Tailoring | Establish individualized goals and plan; prioritize goals. Continually discuss/negotiate end point. |
| Modifications | Evaluate plan and modify as needed. |
| How well | Future evaluative efforts will focus on studying the process of coaching and lead to an RCT of the peer system navigation intervention. |
